# EBUS-GS with the GeneXpert MTB/RIF assay for diagnosis of *Mycobacterium tuberculosis* infection of isolated pulmonary nodules

**DOI:** 10.1186/s40001-023-01331-z

**Published:** 2023-09-23

**Authors:** Jie Cao, Ye Gu, Xiao-cui Wu, Li-ping Cheng, Lei Wang, Qing-rong Qu, Wei Sha, Qin Sun

**Affiliations:** 1 Shanghai Clinical Research Center for Infectious Disease (Tuberculosis), Shanghai Key Laboratory of Tuberculosis, Shanghai Pulmonary Hospital, School of Medicine, Tongji University, Shanghai, China; 2 Department of Bronchoscopy, Shanghai Pulmonary Hospital, School of Medicine, Tongji University, Shanghai, China; 3 Department of Clinical Laboratory, Shanghai Pulmonary Hospital, School of Medicine, Tongji University, Shanghai, China

**Keywords:** EBUS-GS, Xpert MTB/RIF, Tuberculoma, Isolated pulmonary nodules, Diagnosis

## Abstract

**Objective:**

Investigate the use of endobronchial ultrasonography with a guide sheath (EBUS-GS) combined with Gene Xpert MTB/RIF (Xpert) for diagnosis of *Mycobacterium tuberculosis* (MTB) infection in isolated pulmonary nodules.

**Methods:**

Patients who had isolated pulmonary nodules and unknown diagnoses at our institution from October 2020 to December 2021 were prospectively examined using EBUS-GS and Xpert. The diagnostic values of using EBUS-GS or bronchoalveolar lavage fluid (BALF) with acid-fast staining, MGIT 960 culture, pathological examination, and Xpert for isolated pulmonary nodules caused by MTB infection were compared using receiver operating characteristic (ROC) analysis.

**Results:**

There were 135 patients, 64 with isolated pulmonary tuberculomas and 71 with non-tuberculous lesions. The sensitivity of EBUS-GS with Xpert was significantly higher than BALF with Xpert (57.81% vs. 34.78%, P = 0.017). Use of EBUS-GS with Xpert and MGIT 960 culture further increased the sensitivity to 62.50% (95%CI 50.64–74.36) and increased the specificity to 100%. The AUC values of BALF with MGIT 960 culture was 0.663(95%CI 0.543–0.783) and BALF with Xpert was 0.674 (95%CI 0.556–0.792). The AUC values of EBUS-GS with MGIT 960 culture was 0.680 (95%CI 0.554–0.743), with pathological examination was 0.713 (95%CI 0.573–0.760), and with Xpert was 0.789 (95%CI 0.655–0.829).

**Conclusion:**

Use of EBUS-GS with Xpert had high sensitivity and specificity in the diagnosis of isolated pulmonary tuberculoma. This method has significant potential for use in clinical practice.

## Introduction

Tuberculosis (TB) is a significant global public health problem. The 2021 Global Tuberculosis Report of the World Health Organization (WHO) concluded that about 9.9 million people were suffering from TB and 1.3 million died from TB during 2020. China had 841,500 newly diagnosed TB patients in 2020, accounting for 8.5% of the global total, second only to India [1]. Early diagnosis and treatment are keys for reducing the burden of TB [2]. The positive detection of pathogens in sputum (molecular testing, culture) is considered the gold standard for diagnosing tuberculosis [3]. However, a 2019 study reported that smear-negative pulmonary TB accounted for approximately 70% of all cases of pulmonary TB in China, and this was the main reason for the slow decline in the prevalence of pulmonary TB [4].

Pulmonary tuberculoma is a special type of secondary pulmonary TB that has no characteristic clinical manifestations, but has imaging manifestations of atypical non-calcified solitary pulmonary nodules [5]. Pulmonary tuberculoma can be easily confused with peripheral lung cancer, pulmonary sarcoidosis, benign lung tumor, and other conditions [6–8]. Patients with pulmonary tuberculoma have extremely low rates of smear-positive and culture-positive sputum [9]. Samples obtained by minimally invasive examinations may improve the diagnostic efficiency based on pathological and etiological detection [10]. Clinicians commonly perform percutaneous pulmonary puncture for biopsy because this method is simple, minimally invasive, and inexpensive. However, this method is also less precise when the lesion is tiny or located at the inner one-third of lung, and can also lead to pneumothorax and bleeding [11]. It is often difficult for a clinician using a common bronchoscope to reach peripheral pulmonary lesions, so this method often fails to obtain a sufficient amount of tissue for the confirmation of bacteriologically-negative TB.

Endobronchial ultrasonography (EBUS) is an efficient and minimally invasive interventional bronchoscopic technique that is increasingly used because of its good performance in the diagnosis of pulmonary diseases, such as tumors [12–14]. EBUS with a guide-sheath (EBUS-GS) uses a small-diameter, annular ultrasound probe that is inserted into a guide sheath. Clinicians can use EBUS-GS to enter a grade 8 to 9 bronchus, and then use the GS to establish a channel for biopsy under ultrasound guidance [14, 15]. There are few studies on EBUS-GS in the diagnosis of isolated pulmonary nodules caused by *Mycobacterium tuberculosis* (MTB) infection [14]. Due to the limited tissue obtained by EBUS-GS, it is still difficult to diagnose TB by pathological examination alone. To improve diagnostic efficiency, it is necessary to combine EBUS-GS with a rapid and effective pathogen detection method, such as GeneXpert MTB/RIF (Xpert; Cepheid, Sunnyvale, CA, USA), a cartridge-based, automated hemi-nested real time PCR system that uses five overlapping molecular beacon probes (A–E) which target the rifampicin resistance determining region (RRDR) of the *rpoB* gene [16]. This method thus allows detection of *Mycobacterium tuberculosis* complex (MTBC) and mutations conferring rifampicin resistance within 2 h [17]. The Xpert system is now widely used for the clinical diagnosis of pulmonary TB [18]. In this study, we analyzed the diagnostic value of EBUS-GS combined with Xpert for identification of MTB infections in isolated pulmonary nodules.

## Materials and methods

### Study design and patient enrollment

This study selected patients with isolated pulmonary nodules and unclear diagnosis who were admitted to Shanghai Pulmonary Hospital, Tongji University affiliated, from October 2020 to December 2021. Case selection was based on inclusion criteria. All enrolled cases underwent comprehensive diagnostic examinations, including complete blood count, blood biochemistry, coagulation profile, QuantiFERON-TB Gold (QFT) test, rheumatologic immune markers, tumor markers, etc. After excluding contraindications such as bleeding tendency, acute coronary syndrome, severe hypertension, severe ventilatory dysfunction, etc., the patients underwent endobronchial ultrasound-guided transbronchial lung biopsy (EBUS-GS) under local anesthesia and bronchoalveolar lavage (BAL) under bronchoscopy. EBUS-GS was performed first, followed immediately by BAL. EBUS-GS biopsy samples were subjected to smear, culture, Xpert, liquid-based cytological test, and histopathological examinations, while BAL fluid samples were subjected to smear, culture, Xpert, and liquid-based cytological test. Patients diagnosed with tuberculoma were classified as the TB group, while those diagnosed with other diseases were classified as the non-TB pulmonary disease group. This study was approved by the Ethics Review Committee of Shanghai Pulmonary Hospital affiliated to Tongji University (Ethics No. K20-009). All patients signed informed consent documents prior to bronchial examination and biopsy.

The inclusion criteria were: (*i*) patient age from 16 to 80-years-old; (*ii*) at least two negative results for acid-fast bacilli and at least one negative molecular detection result for TB in sputum; (*iii*) isolated pulmonary nodules (≥ 10 mm and ≤ 30 mm in diameter) based on imaging of pulmonary lesions; and (*iv*) HIV negativity. The exclusion criteria were: (i) patient inability to cooperate; (ii) pulmonary nodules smaller than 10 mm in diameter, inability of the bronchoscope to reach the lesion, or failure of the EBUS-GS procedure; or (iii) unclear final diagnosis; or (iv) loss to follow up.

### Diagnostic criteria for pulmonary tuberculoma

The diagnosis of pulmonary tuberculoma was based on the guidelines of ATS/CDC/IDSA [19] and the Health Industry Standards of the People's Republic of China Diagnosis of Tuberculosis (WS 288-2017), which was issued by the National Health Commission on May 1, 2018.

A patient diagnosed with bacteriologically or histopathologically confirmed TB had any one or more of the following criteria: (i) positive for MTB based on Bectec MGIT 960 culture in sputum, bronchoalveolar lavage fluid (BALF), or puncture fluid; (ii) positive for MTB based on molecular tests of BALF or puncture fluid; (iii) positive for MTB based on histopathological examination of puncture, biopsy, or surgical samples with granulomatous inflammation and epithelioid cells, caseous necrosis, and Langerhans cells in the lesions observed by microscopy, with positive TB-DNA-PCR results, with or without positive results from acid-fast staining.

A patient clinically diagnosed with TB met all the following criteria: (i) positive in the interferon gamma (IFN-γ) release test; (ii) exclusion of non-tuberculous pulmonary diseases; (iii) efficacy of anti-TB treatment.

### Experimental methods

The EBUS-GS used a xenon light source and image processing station (Olympus, CV-260SL), a flexible bronchoscope (Olympus, BF-1T260), an ultrasonic endoscope system (Olympus, EU-ME1), and a bronchial ultrasonic probe (Olympus, UM-S30-20R).

Bronchoscopic alveolar lavage was performed basically based on the 2017 Chinese expert consensus on pathogen detection in bronchoalveolar lavage for infectious pulmonary diseases, published by the Chinese Medical Association, and the 2012 ATS clinical practice guideline [20]. One associate chief physician specializing in respiratory endoscopy was responsible for the bronchoscopy procedures in this study. After fasting and water-deprivation for 4 h, an anesthesia spray (2% lidocaine) was administered while the patient was asked to breathe in, making the glottis and larynx fully anesthetized. Ten minutes later, an electronic bronchoscope was introduced through the nose or mouth, which was accompanied by lidocaine spray (2%, 10 mL) of the vocal cords. After the bronchoscope reached the trachea, 2% lidocaine was injected into the main airway and carina to reduce the airway mucosal reflex. Then the bronchoscope entered the opening of the main bronchial tube, each lobe, and segmental bronchus, from top to bottom. Finally, the locations of lesions were determined according to the imaging findings and bronchoscopic manifestations. The bronchoscope tip was positioned in a wedge-shaped position within the target bronchial segment. A rapid instillation of at least 20 mL of room temperature sterile saline solution was administered through the working channel. Immediately after instillation, negative pressure suction was applied to obtain the lavage fluid. This process was repeated two times. The BALF specimens were obtained and examined by acid-fast staining, MGIT 960 culture, Xpert, and a liquid-based cytological test.

**Fig. 1:**
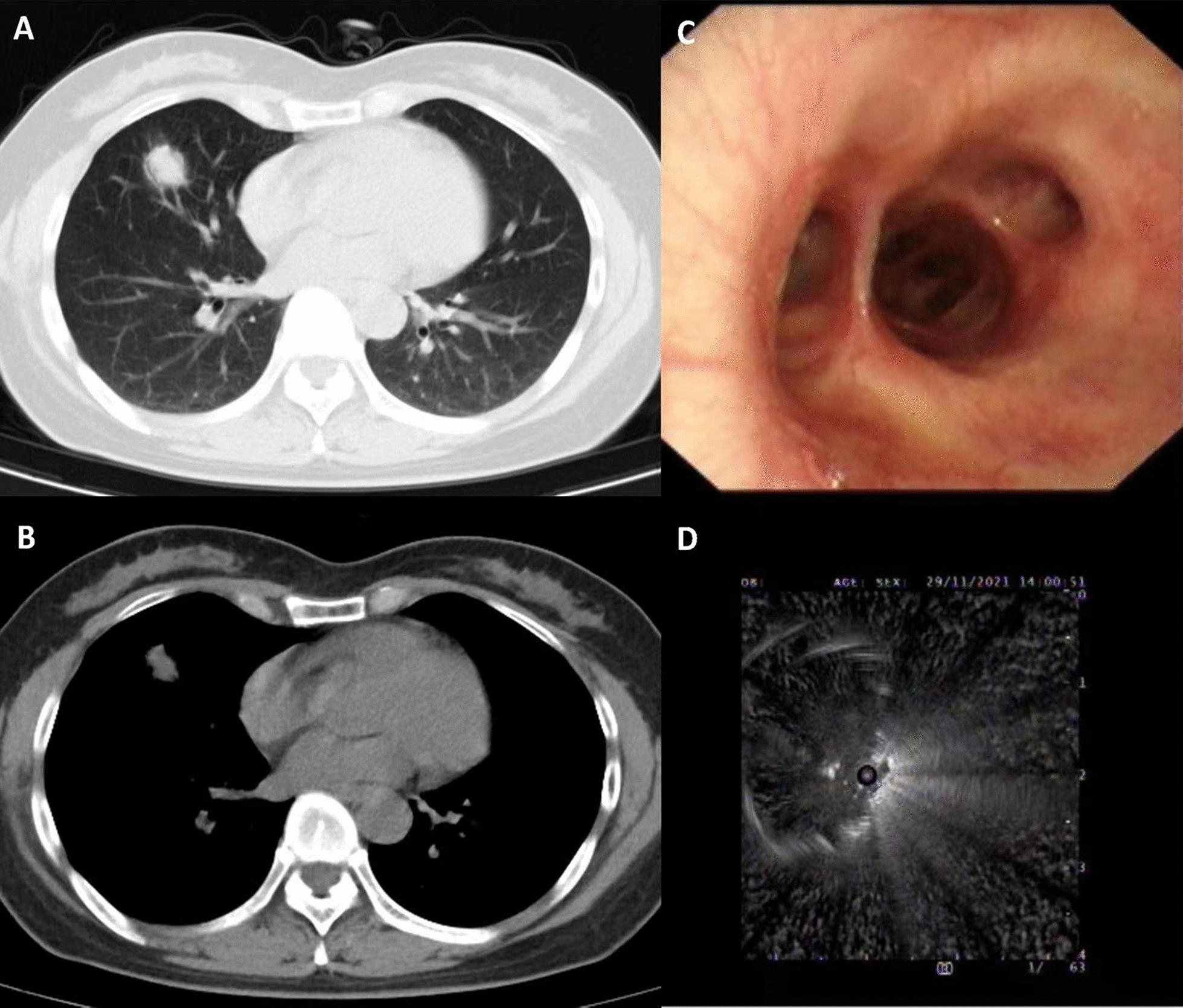
Case 1, a 45-year-old female who was diagnosed with pulmonary tuberculosis in the middle lobe of the right lung. **A**,** B** Chest CT, showing of lesions in the lung window and mediastinal window. **C**,** D** Ultrasound from EBUS-GS, showing lesions around the ultrasound probe

**Fig. 2:**
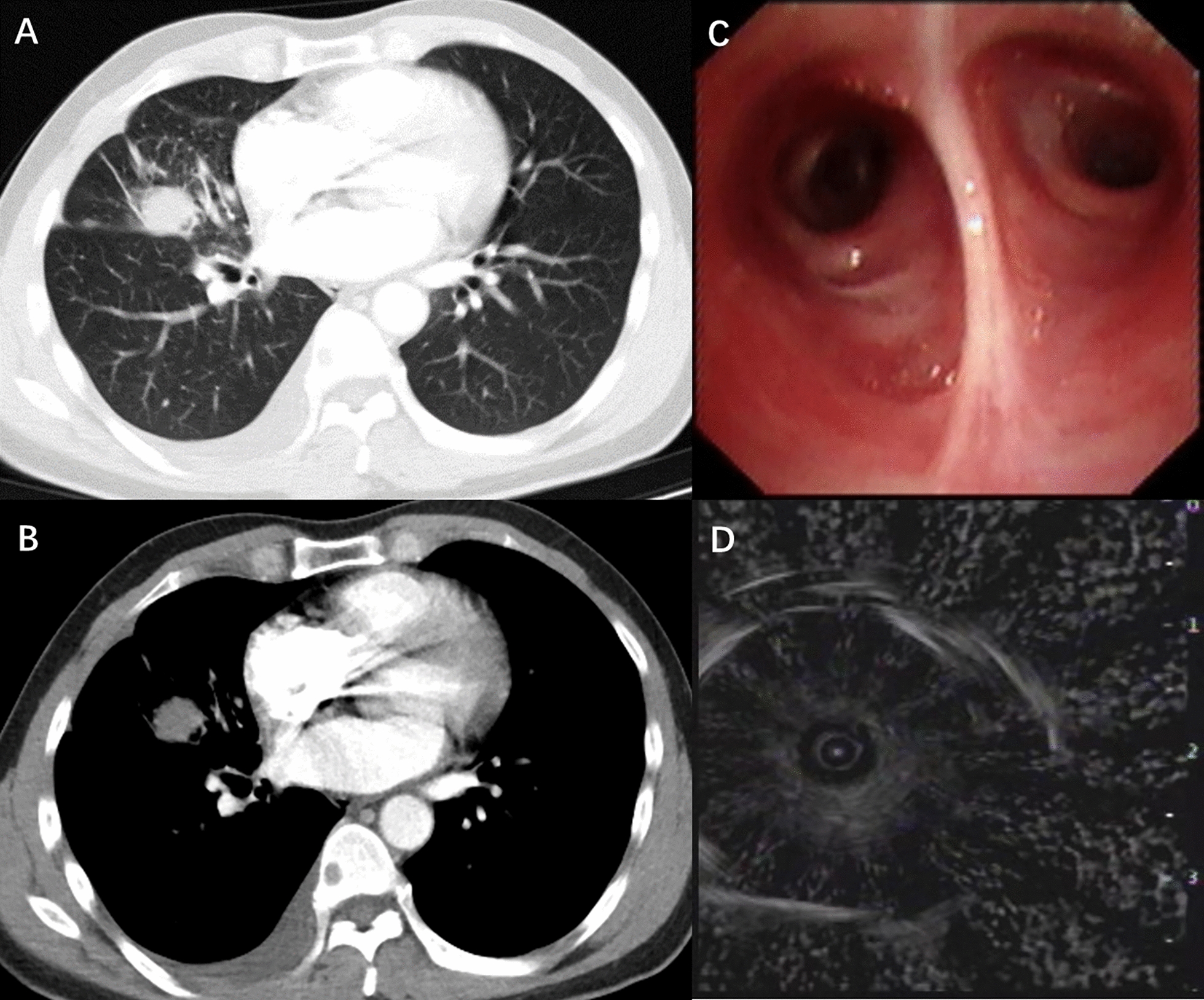
Case 2, a 42-year-old man who was diagnosed with non-small-cell lung cancer in the middle lobe of the right lung. **A**, **B** Chest CT, showing lesions in the lung window and mediastinal window. **C**, **D** Ultrasound from EBUS-GS, showing lesions around the ultrasound probe

For EBUS-GS, the patient was given a local anesthesia, and then placed in a supine position on the examination bed. First, a routine bronchoscopy was performed to observe the mucosa and lumen of the bronchus. Then, the ultrasound probe was sent to the distal end of the bronchial branch of the lesion until it could not go further. The bronchus was scanned to determine the location of the lesion and the surrounding blood vessels. After the site for puncture was determined, the guiding sheath was fixed, and the ultrasonic probe was withdrawn. All lesions (3–5 samples per patient) were obtained using biopsy forceps and cell brushes and were routinely sent for pathological examination. At the end of biopsy and sampling, 10 mL of normal saline was injected into the guide sheath and recovered using negative pressure suction for further etiological examination (Figs. 1 and 2).

For acid-fast staining and Bactec MGIT 960 culturing, specimens were first smeared and dried. Then, acid-fast staining was performed, followed by microscopic examination. The BACTEC MGIT 960 automatic analyzer (BD, USA) was used for rapid culture of *Mycobacterium* according to the instructions of the manufacturer.

For pathological examination, biopsy specimens were fixed by formalin, embedded in paraffin, and then sectioned. Then hematoxylin–eosin staining and acid-fast staining were performed. Pathological diagnosis was based on the histopathological and immunohistochemical results. Real-time fluorescence quantitative PCR was used to detect specific MTB DNA fragments: IS6110 [21], RV0577, and 16S rRNA.

For the Xpert assay, sterile normal saline (1 mL) was added to the tissue samples, followed by centrifugation at 3000 rpm for 15 min. Then the supernatant was discarded and the remaining precipitate was used for the Xpert test. The automatic GeneXpert was used to detect positive MTB and Xpert (Cepheid, USA) was used to test resistance to rifampin according to the instructions of the manufacturers.

### Statistical analysis

All data were analyzed using SPSS version 24.0 (SPSS Inc., Chicago, IL, USA). Continuous variables with normal distributions were expressed as means ± standard deviations (SDs) and groups were compared using Student’s *t-*test. Categorical data were compared using the Chi-square test. The values of EBUS-GS and BALF when used with acid-fast staining, MGIT 960 culture, pathological examination, and Xpert for the diagnosis of pulmonary tuberculoma were compared by calculation of sensitivity, specificity, positive predictive value (PPV), negative predictive value (NPV), positive likelihood ratio (PLR), negative likelihood ratio (NLR), and Youden's index. For each test, a receiver operating characteristic (ROC) curve was drawn, and the area under the curve (AUC) was calculated. All tests were two-sided, and P value below 0.05 was considered statistically significant.

## Results

### Baseline demographic and clinical characteristics of enrolled patients

**Fig. 3:**
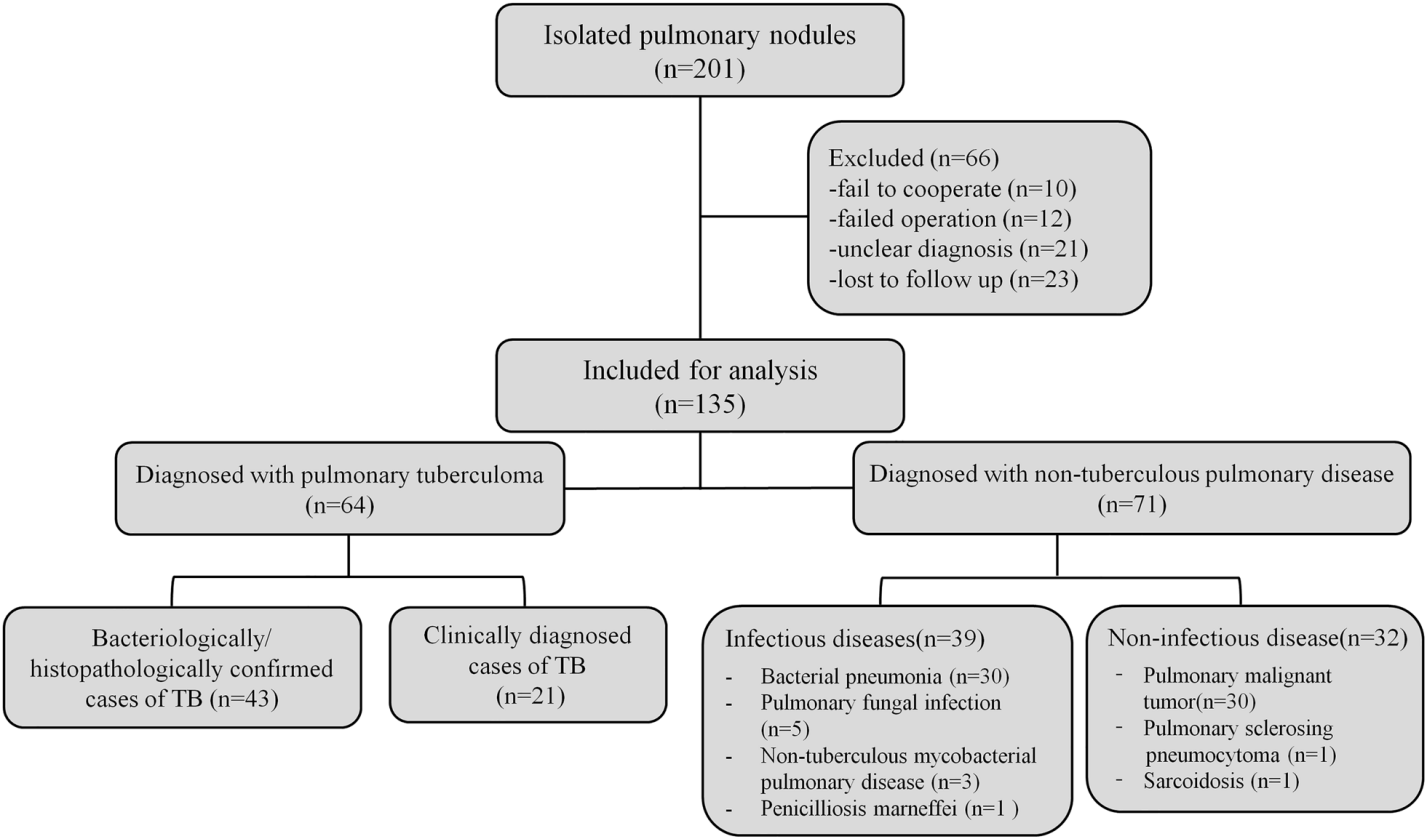
Disposition of 201 patients who were admitted for isolated pulmonary nodules between October 2020 and December 2021

We initially examined isolated pulmonary nodules from 201 patients who were admitted to the Tuberculosis Department between October 2020 and December 2021 (Fig. 3). We excluded 66 patients based on our predefined exclusion criteria, and included 135 patients in the final analysis. Sixty-four patients had isolated pulmonary tuberculomas (43 with bacteriological or histopathological confirmation and 21 with clinical confirmation) and 71 patients had non-tuberculous diseases (39 with infectious diseases and 32 with non-infectious diseases).

We initially compared the baseline characteristics of the TB and non-TB groups (Table 1). Overall, the mean age was 55.80 ± 14.07 years, and there were 95 males (70.37%) and 40 females (29.63%). The two groups did not differ in gender, age, BMI, symptoms, smoking, diabetes, symptoms, the size and location of the lesion. However, the TB group had a greater positivity in the QuantiFERON-TB (QFT) assay (90.63% *vs.* 49.30%, P < 0.001).

**Table 1:** Baseline demographic and clinical characteristics of patients who had pulmonary tuberculoma or non-tuberculous pulmonary diseases

Characteristic	Number of cases n = 135	TB group n = 64	Non-TB group n = 71	P-value
Age (years)	55.80 ± 14.07	53.80 ± 15.09	57.61 ± 12.92	0.117
Gender (n, %)				
Male	95 (70.37%)	43 (67.19%)	52 (73.24%)	0.442
Female	40 (29.63%)	21 (32.81%)	19 (26.76%)	
BMI (kg/m^2^)	21.35 ± 3.65	21.24 ± 3.73	21.81 ± 3.58	0.740
Smoking (n, %)	48 (35.56%)	27 (42.19%)	21 (29.58%)	0.126
Diadetes (n, %)	27 (20.00%)	17 (26.56%)	10 (14.08%)	0.070
Symptoms (n, %)				
Cough	63 (46.67%)	29 (45.31%)	34 (47.89%)	0.765
Chest pain	12 (8.89%)	6 (9.38%)	6 (8.45%)	0.851
Hemoptysis	15 (11.11%)	4 (6.25%)	11 (15.49%)	0.088
Fever	12 (8.89%)	5 (7.81%)	7 (9.86%)	0.677
No symptoms	44 (32.59%)	21 (32.81%)	23 (32.39%)	0.959
QFT (n, %)				
Positive	93 (68.89%)	58 (90.63%)	35 (49.30%)	< 0.001
Negative	42 (31.11%)	6 (9.38%)	36 (50.70%)	
Lesion size (n, %)				
1–2 cm	53 (39.26%)	22 (34.38%)	31 (43.66%)	0.270
2–3 cm	82 (60.74%)	42 (65.62%)	40 (56.34%)	
Lesion location (n, %)				
Right upper lobe	51 (37.77%)	26 (40.63%)	25 (35.21%)	0.534
Right middle lobe	12 (8.89%)	5 (7.81%)	7 (9.86%)	
Right inferior lobe	25 (18.53%)	9 (14.06%)	16 (22.54%)	
Left upper lobe	31 (22.96%)	14 (21.87%)	17 (23.94%)	
Left inferior lobe	16 (11.85%)	10 (15.63%)	6 (8.45%)	

*BMI* body mass index; *QFT* QuantiFERON-TB Gold In-Tube test

### Use of EBUS-GS with Xpert for diagnosis of pulmonary tuberculoma

We performed EBUS-GS in all 135 patients, and then examined the samples using different methods (Table 2). The results showed that EBUS-GS with acid-fast staining had a sensitivity of 17.19% and a specificity of 100%; EBUS-GS with MGIT 960 culture had a sensitivity of 35.94% and a specificity of 100%; EBUS-GS with pathological examination had a sensitivity of 46.88% and a sensitivity of 95.77%; and EBUS-GS with Xpert had a sensitivity of 57.81% and a specificity of 100%. When EBUS-GS was combined with Xpert and MGIT 960 culture, the sensitivity was 62.50% and the specificity was 100%. When EBUS-GS was combined with Xpert and pathological examination the sensitivity was 62.50% and the specificity was 95.77%.

**Table 2:** Diagnostic value of using BALF or EBUS-GS with acid-fast staining, MGIT 960 culture, pathological examination, and Xpert for solitary pulmonary tuberculoma

Clinical specimen	Sensitivity% (95%CI)	Specificity% (95%CI)	PPV% (95%CI)	NPV% (95%CI)	PLR (95%CI)	NLR (95%CI)	YI (95%CI)
BALF + acid-fast staining	8.69 (4/46)	100 (30/30)	100 (4/4)	41.67 (30/72)	–	0.91	0.09
	0.55–16.84	100–100	100–100	30.28–53.05		0.84–0.99	0.00–0.17
BALF + MGIT 960	32.61 (15/46)	100 (30/30)	100 (15/15)	49.18 (30/61)	–	0.67	0.33
	19.06–46.16	100–100	100–100	36.63–61.73		0.55–0.82	0.19–0.46
BALF + Xpert	34.78 (16/46)	100 (30/30)	100 (16/16)	50.00 (30/60)	–	0.65	0.35
	21.02–48.55	100–100	100–100	37.35–62.65		0.53–0.81	0.21–0.49
BALF + Xpert + MGIT 960	36.96 (17/46)	100 (30/30)	100 (17/17)	50.85 (30/59)	–	0.63	0.37
	23.01–50.91	100–100	100–100	38.09–63.60		0.51–0.79	0.23–0.51
EBUS-GS + acid-fast staining	17.19 (11/64)	100 (71/71)	100 (11/11)	57.26 (71/124)	–	0.83	0.17
	7.94–26.43	100–100	100–100	48.55–65.97		0.74–0.93	0.08–0.26
EBUS-GS + MGIT 960	35.94 (23/64)	100 (71/71)	100 (23/23)	63.39 (71/112)	–	0.64	0.36
	24.18–47.69	100–100	100–100	54.47–72.31		0.53–0.77	0.24–0.48
EBUS-GS + pathological examination	46.88 (30/64)	95.77 (68/71)	90.91 (30/33)	66.67 (68/102)	11.09 3.56–34.61	0.55	0.43
	34.65–59.10	91.10–100	81.10–100	57.52–75.82		0.44–0.70	0.30–0.56
EBUS-GS + Xpert	57.81 (37/64)	100 (71/71)	100 (37/37)	72.45 (71/98)	–	0.42	0.58
	45.71–69.91	100–100	100–100	63.60–81.29		0.32–0.56	0.46–0.70
EBUS-GS + Xpert + MGIT 960	62.50 (40/64)	100 (71/71)	100 (40/40)	74.74 (71/95)	–	0.38	0.63
	50.64–74.36	100–100	100–100	66.00–83.47		0.27–0.51	0.51–0.74
EBUS-GS + Xpert + pathological examination	62.50 (40/64)	95.77 (68/71)	93.02 (40/43)	73.91 (68/92)	14.79 4.81–45.50	0.39	0.58
	50.64–74.36	91.10–100	85.41–100	64.94–82.89		0.28–0.54	0.46–0.71

*BALF* bronchoalveolar lavage fluid; *MGIT* Mycobacteria Growth Indicator Tube; *EBUS-GS* endobronchial ultrasound with guide-sheath; *PPV* positive predictive value; *NPV* negative predictive value; *PLR* positive likelihood ratio; *NLR* negative likelihood ratio; *YI* Youden index; *CI* confidence interval

We also performed bronchoalveolar lavage and collected BALF in 76 patients (56.30%; Table 2). In the remaining 59 cases, due to complications such as bleeding or an inability to tolerate prolonged procedure time, only a simple microscopic observation was performed after completing the EBUS-GS examination. Analysis of the BALF samples indicated acid-fast staining had a sensitivity of 8.69% and a specificity of 100%; MGIT 960 culture had a sensitivity of 32.61% and a specificity of 100%; and Xpert had a sensitivity of 34.78% and a specificity of 100%. When combined Xpert with MGIT 960 culture for BALF, the sensitivity was 36.96%, and the specificity was 100%.

In this study, EBUS images were categorized into two groups: 1) within (94 cases) and 2) adjacent to (41 cases). Among the 94 cases, the sensitivity of EBUS GS + Xpert diagnosis was 61.5% (24/39). Although this sensitivity was higher than the 52% (13/25) observed in the 41 cases, the difference was not statistically significant (P = 0.451). The specificity in both groups was 100%.

### Consistency of the different methods

**Fig. 4:**
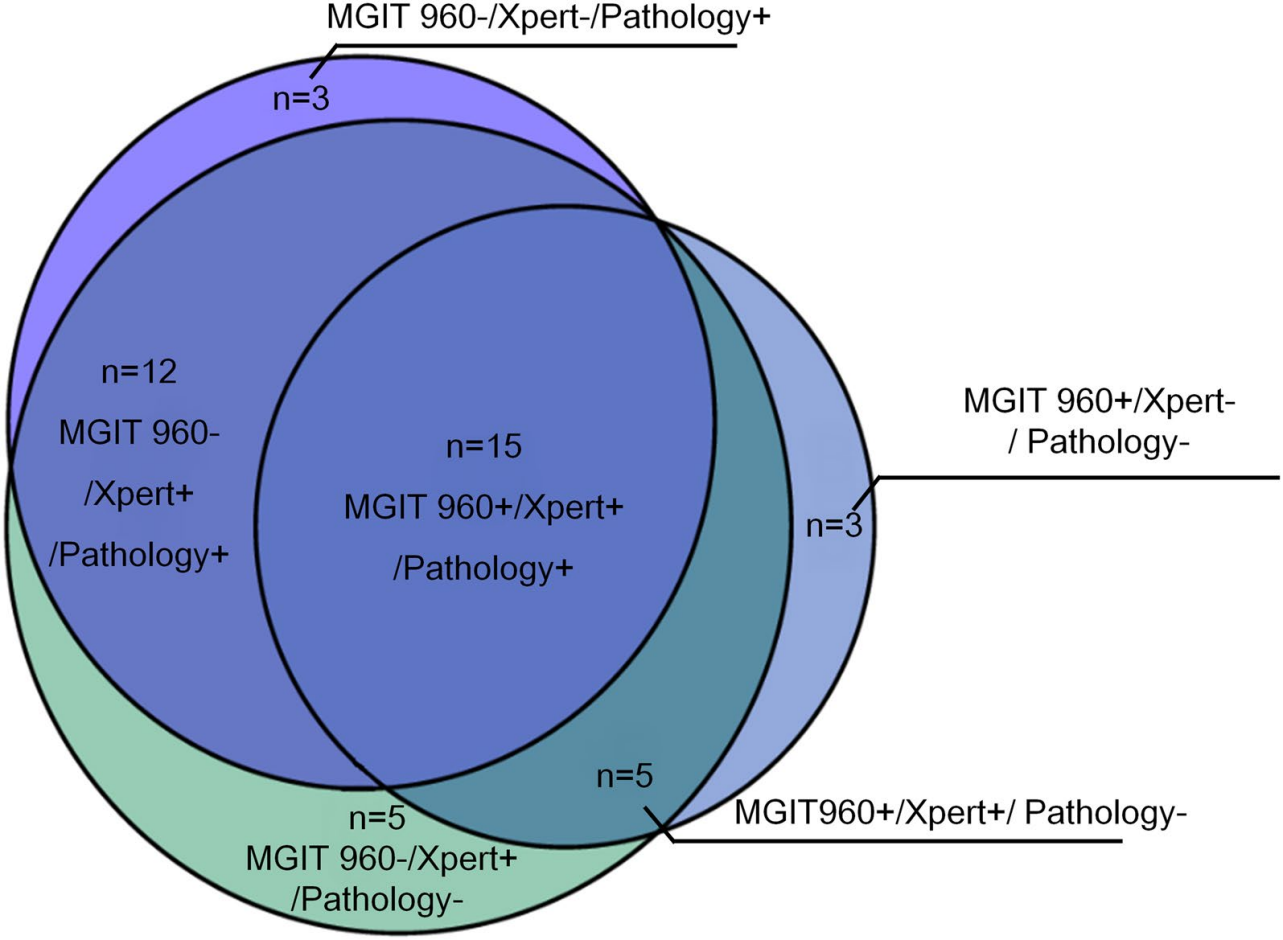
Consistency of diagnosis from EBUS-GS with MGIT 960, Xpert MTB/RIF and pathological examination

We then analyzed all 43 bacteriologically/histopathologically confirmed cases of TB based on EBUS-GS (Fig. 4). Fifteen were positive for MGIT 960 culture with Xpert and with pathological examination, 12 were positive for Xpert with pathological examination, 5 were positive for MGIT 960 culture with Xpert, 5 were positive for Xpert alone, and 3 were positive for pathological examination alone, and 3 were positive for MGIT 960 culture alone.

### Diagnostic sensitivity of the different methods

The sensitivity of EBUS-GS with Xpert was significantly higher compared to BALF with Xpert (57.81% *vs.* 34.78%, P = 0.017). The sensitivity of EBUS-GS with MGIT 960 culture (35.94%) was higher compared to BALF with MGIT 960 culture (32.61%); and the sensitivity of EBUS-GS with acid-fast staining (17.19%) was higher compared to BALF with acid-fast staining (8.69%). However, there was no statistical significance between them (P = 0.717 and 0.200, respectively).

### ROC analysis of the different methods

**Fig. 5:**
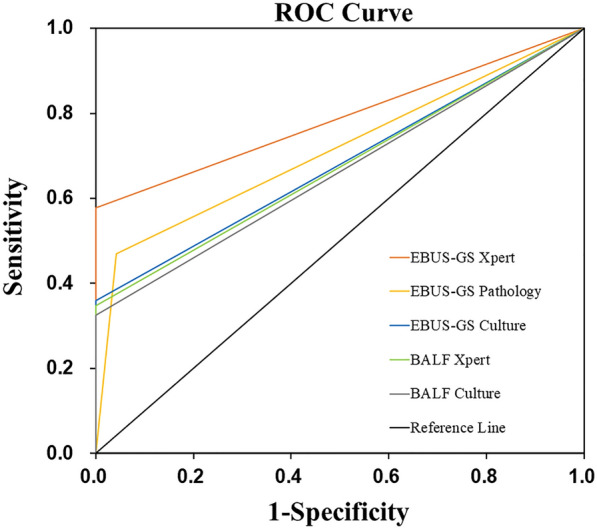
ROC curves for BALF with MGIT 960 culture or Xpert and of EBUS-GS with MGIT 960 culture, pathological examination, or Xpert

We compared the AUC values of the different methods (Fig. 5). BALF with MGIT 960 culture had an AUC of 0.663 and BALF with Xpert had an AUC of 0.674. EBUS-GS with MGIT 960 culture had an AUC of 0.680, EBUS-GS with pathological examination had an AUC of 0.713, and EBUS-GS with Xpert had an AUC of 0.789.

## Discussion

This study examined lesions of suspected pulmonary tuberculoma based on the characteristics of isolated nodules. Our major result was that the diagnostic sensitivity of EBUS-GS was significantly higher than that of BALF when used with Xpert, MGIT 960 culture, and acid-fast staining. Our results indicated that EBUS-GS with Xpert had the highest sensitivity and specificity for the diagnosis of pulmonary tuberculoma among all tested methods. This method therefore has significant value for use in clinical practice.

Previous research reported that the etiological diagnosis of smear-negative pulmonary TB was difficult [22]. Khan et al. found that the positive rate of acid-fast staining in BALF was only 25.9%, although the positive rate of BALF with culture reached 63% [23]. Zheng et al. performed a retrospective analysis and reported the sensitivity of BALF with Xpert was 68.8% [3]. Compared with smear-negative pulmonary TB, the diagnosis of pulmonary tuberculoma with isolated nodules is more difficult, resulting in a lower detection rate. Thus, previous studies found that 57.1% to 92% of TB patients in areas with endemic TB were misdiagnosed as having primary lung cancer [24]. It is common for some patients with pulmonary tuberculoma who were misdiagnosed as lung cancer and then received surgery in clinical practice.Therefore, a more accurate and minimally invasive examination is needed to obtain specimens and improve the diagnosis of pulmonary tuberculoma from isolated nodules.

Bronchoendoscopy techniques have developed rapidly during recent years. Ordinary bronchoendoscopy has limited value in the diagnosis of isolated pulmonary nodules [25]. The advantages of EBUS-GS are that it uses an ultra-fine bronchoscope and an annular ultrasound probe. As a minimally invasive diagnostic technology, EBUS-GS has greater flexibility, it leads to fewer complications, it can reach lesions around the central airway, and it can be used to guide entry into peripheral pulmonary lesions. The ultrasonic probe in EBUS-GS can help clinicians to avoid blood vessels in the surrounding regions, and thus improve safety and accuracy [26]. EBUS-GS has been used in clinical practice and in the differential diagnosis of peripheral pulmonary lesions [27], especially in the diagnosis of lung cancer. Zhu et al. found that the diagnostic rate of EBUS-GS for peripheral lung cancer was 64% in 150 patients, and that the postoperative complications from EBUS-GS were significantly less than those from CT-guided transthoracic needle aspiration [28]. Similarly, Bo et al. studied solitary pulmonary nodules (SPNs) and reported that EBUS-GS significantly increased the diagnostic rate of to 72.3% [15]. However, little is known about the use of EBUS-GS for the diagnosis of isolated pulmonary nodules caused by MTB. The present single-center prospective study demonstrated that the sensitivity when EBUS-GS was used with acid-fast staining, MGIT 960 culture, and Xpert were significantly higher when BALF was used with these assays. In other words, compared with bronchoalveolar lavage using a bronchoscope, EBUS-GS is better in locating the lesion and obtains more valuable specimens based on puncture and biopsy. Thus, the EBUS-GS greatly improved the diagnostic accuracy of pulmonary TB.

Xpert is an automated real-time nucleic acid amplification technology that provides rapid and simultaneous detection of tuberculosis and rifampicin resistance. Because Xpert MTB/RIF is more efficient and rapid than traditional methods, it is now widely used for the diagnosis of TB [29, 30], and the WHO recommends this method as a first-line rapid test for the diagnosis of pulmonary TB [31]. A previous study reported the sensitivity of BALF with Xpert among smear-negative or sputum-scarce patients with pulmonary TB was 58.9% [32], higher than in our study (34.78%). However, our sensitivity was 57.81% and specificity was 100% when EBUS-GS was used with Xpert. In addition, our AUC for EBUS-GS with Xpert was 0.789, significantly higher than the other methods. Although the sensitivity of EBUS-GS with Xpert in our study was still not ideal, it greatly improved the etiological diagnosis of isolated pulmonary nodules caused by MTB. The combined use of QFT, a PPD skin test, and other examinations makes it much easier to make a correct clinical diagnosis. We also found that EBUS-GS with Xpert and with MIGT 960 culture or pathological examination slightly increased the sensitivity. In the study by Lan Yao et al., ultrasound bronchoscopy combined with Xpert Ultra was used for the diagnosis of smear-negative pulmonary tuberculosis [33]. Xpert Ultra is a new diagnostic technology recommended by the World Health Organization (WHO). It integrates two different multicopy amplification targets, IS6110 and IS1081, and includes a larger DNA amplification chamber. The study found that Xpert Ultra had a higher sensitivity compared to Xpert (78.1% vs 64.6%) [33]. Therefore, combining EBUS-GS with Xpert Ultra technology may further improve the diagnostic sensitivity for isolated pulmonary tuberculosis nodules.

Pathological examination is crucial for the diagnosis of isolated pulmonary tuberculoma, and EBUS-GS can obtain pathological specimens from isolated pulmonary nodules. However, other diseases, such as nontuberculous mycobacterial pulmonary disease (NTM-PD) and granulomatous diseases of the lung, have clinical and pathological characteristics that are similar to pulmonary TB [34, 35]. In this study, the sensitivity of EBUS-GS with pathological examination (46.88%) was unacceptable, although the specificity was 95.77%; there were also 3 patients with false positive results, 1 with sarcoidosis and 2 with NTM-PD. In addition, drug resistance cannot be determined using a pathological examination. However, use of EBUS-GS with Xpert can quickly diagnose TB and confirm the presence of rifampicin resistance, suggesting that this method is more useful in clinical practice.

In general, compared with conventional invasive examinations, EBUS-GS is characterized by smaller wounds and fewer complications, and Xpert has a high diagnostic rate and is relatively rapid. The combined use of these two methods led to good diagnostic efficacy in isolated pulmonary nodules caused by MTB, and was also useful in the differential diagnosis of non-tuberculous pulmonary diseases.

There were some limitations in this study. Firstly, it was a single-center prospective study with a small sample size. Thus, the diagnostic value of EBUS-GS with Xpert for isolated pulmonary nodules caused by MTB needs verification by large multi-center studies. Secondly, the operators of EBUS-GS must have a relatively high level of technical expertise, and considering the angle of the bronchoscope, some lesions may not be reached or puncture failure might occur.

## Conclusion

EBUS-GS combined with Xpert has great clinical value in the diagnosis of isolated pulmonary tuberculoma based on its moderate sensitivity and high specificity. The sensitivity of EBUS-GS is greater when used with Xpert. Thus, use of this method in clinical practice may help to provide early diagnosis pulmonary tuberculoma so that these patients can avoid misdiagnosis and receive prompt treatment.

## Data Availability

The datasets used and/or analyzed are available from the corresponding author on reasonable request.
